# Methotrexate subcutaneous injection for the initial treatment of rheumatoid arthritis can achieve earlier remission compared with oral administration

**DOI:** 10.3892/mi.2026.336

**Published:** 2026-08-11

**Authors:** Yukie Saio

**Affiliations:** Department of Rheumatology, Toho Hospital, Midori, Gunma 379-2311, Japan

**Keywords:** difficult-to-treat rheumatoid arthritis, methotrexate, treatment response, disease-modifying anti rheumatic drugs, rheumatoid arthritis

## Abstract

In Japan, subcutaneous methotrexate (SC-MTX) administration was introduced in 2022; however, available data regarding its use as an initial treatment or its effectiveness when switching from oral MTX remain limited. The present study aimed to examine the introduction timing and effects of a SC-MTX injection in patients with rheumatoid arthritis (RA). The oral MTX administration group (n=202, O-MTX), initial SC-MTX group (n=59, SC-MTX) and SC-MTX-switched group (n=25, SWITCH) were compared for the disease activity score in the disease activity score in 28 joints with C-reactive protein (DAS28-CRP), health assessment questionnaire (HAQ), remission rate of MTX, combination rate of biological agents and JAK inhibitors, the discontinuation rate (DR) of glucocorticoids, alanine aminotransferase, low-density lipoprotein, estimated glomerular filtration rate, hemoglobin, white blood cell count, lymphocyte count, DR of medications. A survey on SC-MTX self-injection was also conducted. SC-MTX achieved greater improvements in both HAQ and DAS28-CRP than O-MTX, and the difference became evident by 12 weeks following treatment introduction. In SWITCH, the disease activity also decreased after switching. The proportions of remission cases on MTX monotherapy at 24 weeks in the O-MTX, SC-MTX and SWITCH groups were 31.3, 65.96 and 33.33%, respectively and the DR rates of glucocorticoids in these groups were 59.3, 85.7 and 50%, respectively. As regards safety, no differences were noted in renal function, liver function, or changes in blood cells. According to the questionnaire for the usability of the SC-MTX self-injection, patients were very satisfied. On the whole, the present study demonstrates that in the treatment of RA, the initial introduction of SC-MTX is considered very useful, particularly in patients with high disease activity.

## Introduction

In 1999, methotrexate (MTX) became available for use in Japan for the treatment of rheumatoid arthritis (RA) (1). In 2011, the upper limit of the dose was extended from 8 to 16 mg per week; however, MTX is only available as an oral medication for the treatment of RA, and numerous patients are unable to take sufficient doses due to side-effects, such as liver dysfunction and gastrointestinal symptoms, such as stomatitis and anorexia (2). In other countries, in the early 2000s, subcutaneous MTX (SC-MTX) injections were reported to be more effective than the oral MTX (O-MTX) administration (3). However, in Japan, it was finally approved as a treatment for RA in September, 2022(1). At Toho Hospital (Midori, Japan), it has been available since March, 2023, and its introduction is currently underway. The 2024 Japan RA Clinical Practice Guidelines Drug Treatment Algorithm states that ‘SC-MTX is expected to be more effective and have equal or greater safety than O-MTX, but O-MTX is prioritized for patients who have not yet been administered MTX due to cost considerations’ (4). However, in countries overseas where MTX is already in circulation, SC-MTX is reported to be as safe as O-MTX, but is more useful than O-MTX (5,6). In addition, the initial introduction of SC-MTX was suggested to have a high efficacy and high continuation rate (5).

Based on the aforementioned findings, the present study aimed to compare the usefulness and safety of the use of O-MTX and SC-MTX injections, the initial administration of SC-MTX, and switching from O-MTX to SC-MTX in patients with RA.

## Patients and methods

### Cases

Among patients who visited the Rheumatology Department of Toho Hospital (Midori, Japan) in June, 2017 and were diagnosed with RA, 202 out of 237 patients who commenced O-MTX treatment at the hospital were observed for 24 weeks, 59 out of 69 patients who were observed for 24 weeks were introduced to SC-MTX, and 25 out of 35 patients who were switched from O-MTX to SC-MTX were observed for 24 weeks (SWITCH group). For all patients, the initial doses were 6 mg/week and 7.5 mg/week for O-MTX and SC-MTX, respectively, based on the Japan College of Rheumatology guideline (7). In cases of switching, treatment was commenced with a dose that did not exceed the O-MTX dose used until then. Subsequently, the O-MTX dose was increased by 2 mg every 4 weeks and SC-MTX by 2.5 mg every 4 weeks, provided that the therapeutic response was insufficient and no adverse effects were observed.

### Research ethics and informed consent

The present study was conducted with the approval of the Institutional Review Board of Toho Hospital (Approval no. 2024-1). In addition, for the analysis of electronic medical record data, informed consent was not obtained; instead, information regarding the use of medical records and possibility of public data presentation was disclosed on the hospital's website (https://www.toho-hp.jp/privacy), in accordance with the Ministry of Health, Labour and Welfare's ‘Ethical Guidelines for Life Sciences and Medical Research Involving Human Subjects’. Patients were provided with adequate opportunity to opt out of participation. Data collection from electronic medical records was initiated from July, 2024. In the survey conducted with patients, written consent was obtained from all participants (Ethics Review Board approval no. 2024-4).

### Evaluation items

The disease activity score in 28 joints with C-reactive protein (DAS28-CRP) and health assessment questionnaire (HAQ) were obtained from electronic medical records prior to administration, and at 4, 12 and 24 weeks following administration to evaluate the therapeutic effect. In addition, the amount of MTX used after 24 weeks, renal function by estimated glomerular filtration rate (eGFR) on initiation and after 24 weeks, glucocorticoid discontinuation rate (DR) after 24 weeks, the concomitant use rate of biological agents and JAK inhibitors after 24 weeks, the remission rate with single agents after 24 weeks, cases of drug discontinuation by 24 weeks and reasons for discontinuation, and anti-cyclic citrullinated peptide (CCP) antibody positivity rate on initiation were examined. Furthermore, to evaluate safety, alanine aminotransferase (ALT), low-density lipoprotein (LDL) and peripheral blood hemoglobin (Hb) levels, white blood cell counts and lymphocyte counts were examined prior to administration, and at 4, 12 and 24 weeks following administration in the SC-MTX groups (SC-MTX and SWITCH groups) for 24 weeks.

### Method for calculating cumulative MTX amount and eGFR change

The cumulative MTX dose was calculated as follows: Cumulative MTX (mg)=weekly dose x total no. of weeks of administration. The change in eGFR was calculated as follows: Change in eGFR=eGFR value at the time of MTX introduction-eGFR value at 24 weeks.

### SC-MTX pen questionnaire

An anonymous questionnaire was administered to investigate patient satisfaction in the group that switched from using syringes to pens (n=43) and the group that had been using pens from the beginning (n=41) (Ethics Review Board approval no. 2024-4). The questionnaire included the age group and sex. For those who had been using the SC-MTX pen from the beginning, the questionnaire items were about: i) The use of the SC-MTX pen; ii) change in anxiety levels about injections since starting to use SC-MTX pen; iii) pain of injections; iv) burden of drug costs (MTX at 8 mg oral administration; 2,388 Yen/month; 30% burden, 716 Yen; SC-MTX 7.5 mg 7,188 yen/month; 30% burden, 2,156 Yen); and v) overall satisfaction with the pen. All were rated on a 10-point scale (higher scores indicate greater satisfaction). The questionnaire administered to the group that switched from using syringes to pens included the following six items, each rated on a 10-point scale (10 points): i) Switching from using a syringe to a pen simplified the operation; ii) switching from using a syringe to a pen reduced fear; iii) switching from using a syringe to a pen increased satisfaction with its use or prompted patients to revert to using a syringe; iv) switching from using a syringe to a pen minimized the pain or induced much more severe pain; v) switching to using a pen increased the medication burden or did not cause a change; and vi) satisfaction with the pen formulation.

### Statistical analysis

Statistical analysis was performed using Bell Curve Excell statistics (ver. 4.08, Social Survey Research Information Co., Ltd., Tokyo Japan; https://bellcurve.jp/ex/). For comparisons among multiple groups, the Kruskal-Wallis test (a non-parametric method) was used. When a significant difference was detected, post hoc multiple comparisons were conducted using the Steel-Dwass test to identify differences between groups. To determine whether an association exhibited between two variables, multiple regression analysis was conducted, and the corresponding correlation coefficient was calculated. Fisher's exact test was used to compare groups regarding the remission rate for 3 groups using EZR software version 1.70. When a significant difference was detected, post hoc multiple comparisons were conducted using the Marascullo's method to identify differences between groups. In order to compare the e-GFR between baseline and 24 weeks following treatment initiation, a paired t-test was utilized. A P-value ≤0.05 was considered to indicate a statistically significant difference.

## Results

### The SC-MTX group could use higher doses than the O-MTX group, and switching to SC-MTX aimed to intensify treatment

A summary of the patient characteristics is presented in Table I. The present study included patients who continued MTX treatment for at least 6 months (202/237 cases in the O-MTX group, 59/69 cases in the SC-MTX group and 25/35 cases in the SWITCH group). The discontinuation rates were 14.77, 14.49 and 28.57% for the O-MTX, SC-MTX and SWITCH groups, respectively.

The ages of the three groups were compared. As demonstrated in Table I, the patients in the O-MTX group had a mean age of 62.0±13.2 years with 71.8% females, the patients in the SC-MTX group had a mean age of 69.1±10.5 years with 67.8% females, and the patients in the SWITCH group had a mean age of 57.4±14.4 years with 67.8% females. Statistical comparisons for age revealed P-values of <0.001 for SC-MTX vs. SWITCH, <0.001 for SC-MTX vs. O-MTX, and 0.2722 for SWITCH vs. O-MTX. These findings suggest that among elderly patients, the SC-MTX group received significantly more initial SC-MTX compared with the other groups. Subsequently, the present study examined the amount of MTX used 24 weeks following the introduction MTX and this was compared between the O-MTX, SC-MTX and SWITCH groups. As demonstrated in Table I, a significantly higher dose could be used in both the SC-MTX and SWITCH groups than in the O-MTX group (O-MTX vs. SC-MTX, P<0.001; O-MTX vs. SWITCH, P<0.001; SC-MTX vs. SWITCH, not significant). As presented in Table I, the anti-CCP antibody positivity rate at the time of introduction was lower in the SC-MTX group than in the other groups. Moreover, switching from O-MTX to SC-MTX in the SWITCH group aimed to intensify treatment, accounting for 16 of 25 cases. In these cases, the introduction of biological disease-modifying antirheumatic drugs or JAK inhibitors was considered, but could not be administered due to economic reasons (data not shown; unpublished observation). In addition, the causes of MTX discontinuation and the ratio of patients receiving treatment with vitamins for stomatitis were evaluated. As demonstrated in Table I, stomatitis and anorexia were observed even in the SC-MTX group, although not as frequently as in the O-MTX group, and no clear difference was observed between the groups. Conversely, when comparing the ratio of patients receiving vitamin B6 to prevent stomatitis, although not to the extent that they were discontinuing treatment, the ratio was significantly higher in the O-MTX group than in the SC-MTX group (P=0.001). These results suggest that SC-MTX can be used at a higher dose than O-MTX and at the same dosage as in cases initially introducing SC-MTX even in cases transitioning from O-MTX to SC-MTX. Among other causes, higher SC-MTX doses could be used in the SC-MTX and SWITCH groups than in the O-MTX group as the emergence of stomatitis was not a concern.

### The initial SC-MTX introduction group exhibits higher disease activity (DAS28-CRP) than the O-MTX group and a higher remission rate than the O-MTX group after 12 weeks

**Figure 1:**
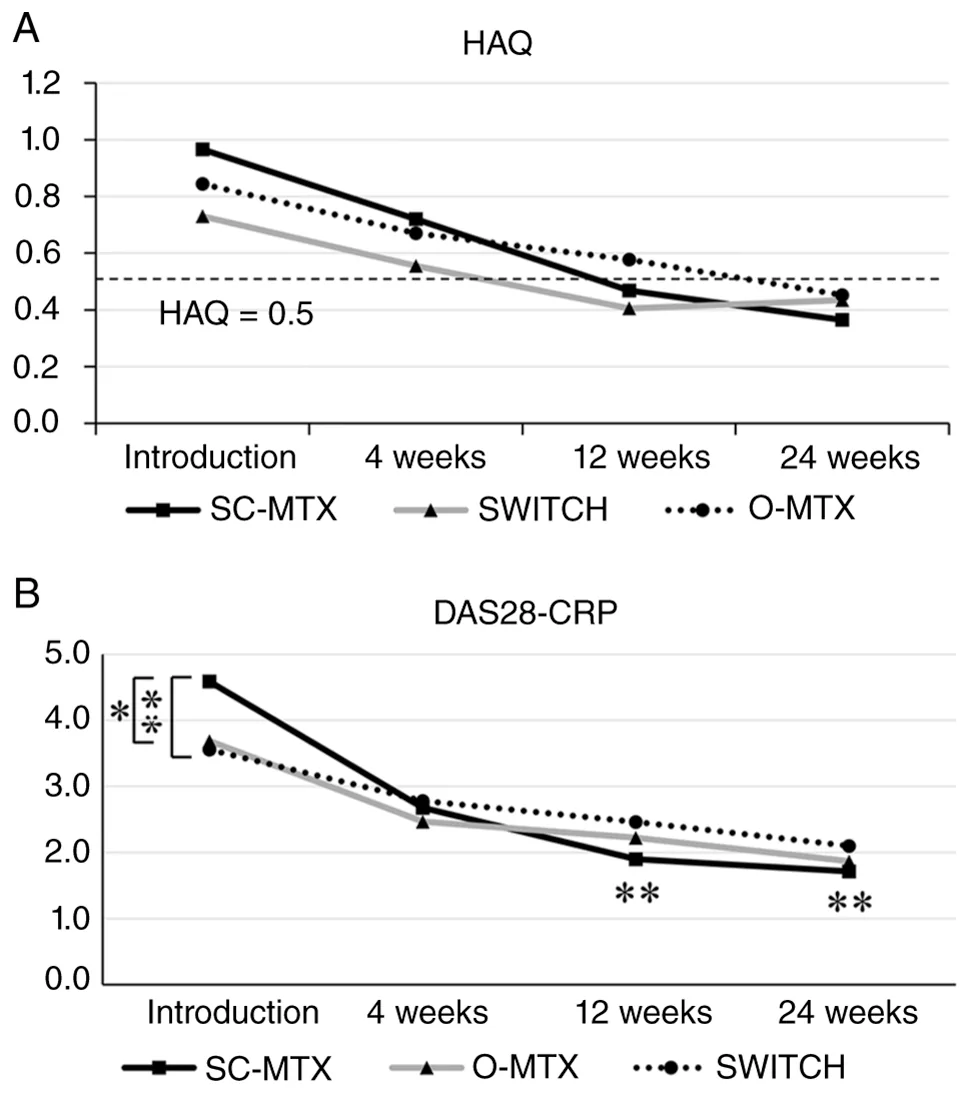
Changes in the HAQ and DAS28-CRP scores in the three groups. (A) Transition of HAQ. The remission threshold for HAQ (0.5) is shown as a thin horizontal dotted line at the 0.5 mark. (B) Transition of DAS28-CRP. ^*^P<0.05, significant differences between the SC-MTX group and the SWITCH group. ^**^P<0.0001, significant differences between the SC-MTX group and the O-MTX group. HAQ, health assessment questionnaire; DAS28-CRP, disease activity score in 28 joints with C-reactive protein; MTX, methotrexate; O-MTX, oral MTX administration; SC-MTX, MTX subcutaneous injection from the initial induction; SWITCH, group switched from O-MTX to MTX subcutaneous injection; W, weeks.

**Figure 2:**
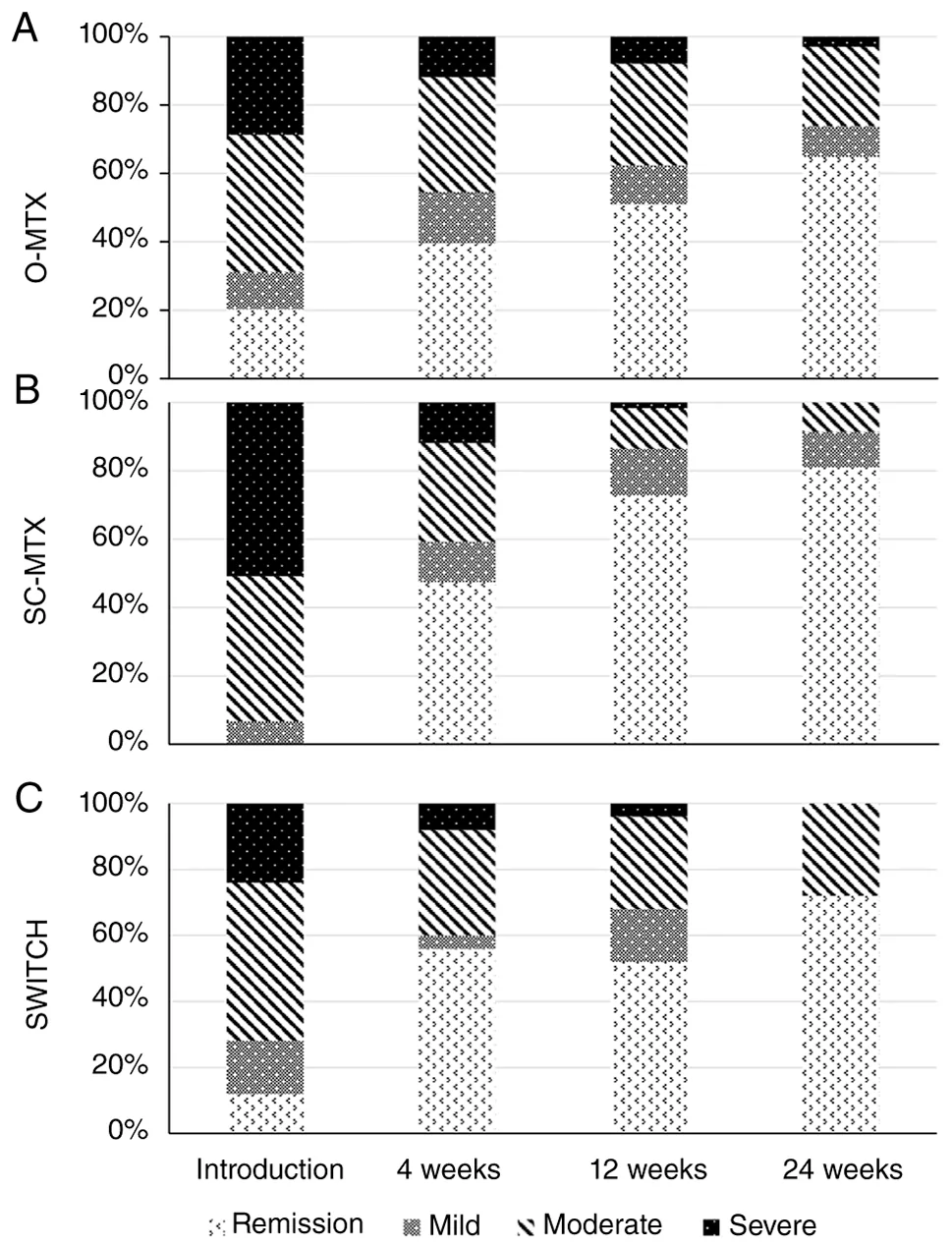
Changes in DAS28-CRP from baseline to 24 weeks in the three groups. The ratios within the groups are displayed according to disease activity: Remission, low disease activity, moderate disease activity and high disease activity. (A) O-MTX group, (B) SC-MTX group, and (C) SWITCH group. DAS28-CRP, disease activity score in 28 joints with C-reactive protein; MTX, methotrexate; O-MTX, oral MTX administration; SC-MTX, MTX subcutaneous injection from the initial induction; SWITCH, switched group from O-MTX to MTX subcutaneous injection; W, weeks.

**Figure 3:**
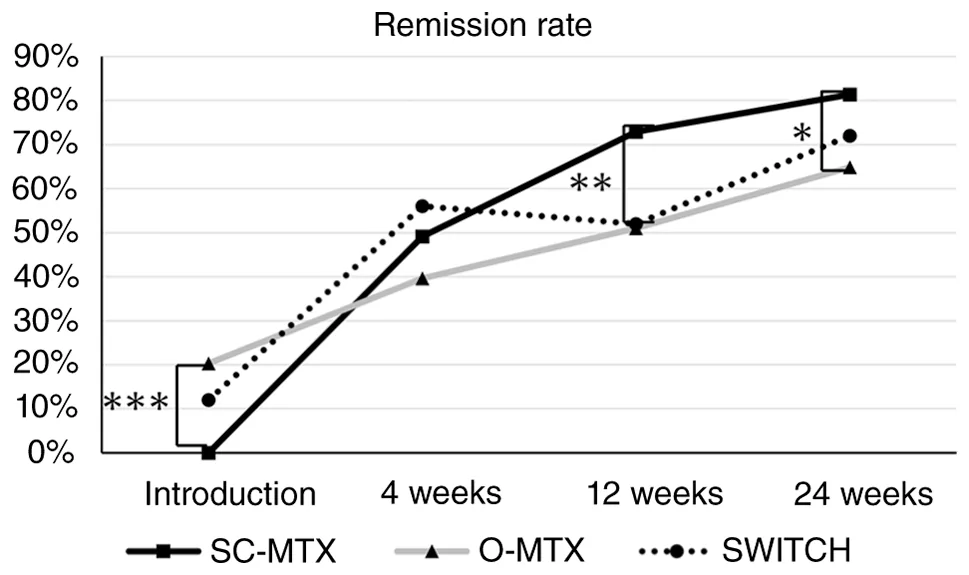
Changes in remission rates based on DAS28-CRP in the three groups. ^*^P<0.05, ^**^P<0.01 and ^***^P<0.001 for significant differences between the SC-MTX group and the O-MTX group. DAS28-CRP, disease activity score in 28 joints with C-reactive protein; MTX, methotrexate; O-MTX, oral MTX administration; SC-MTX, MTX subcutaneous injection from the initial induction; SWITCH, switched group from O-MTX to MTX subcutaneous injection; W, weeks.

Changes in HAQ and DAS28-CRP scores 24 weeks from MTX introduction, during SC-MTX introduction, or on switching to SC-MTX are presented in Fig. 1, and specific values are presented in Table SI. No significant differences in HAQ were observed between any groups at any time points; however, the SC-MTX and SWITCH groups had HAQ scores <0.5 starting from 12 weeks, whereas the O-MTX group only scored <0.5 at 24 weeks (Fig. 1A). Conversely, the initial DAS28-CRP score was significantly higher in the SC-MTX group than in the other groups (SC-MTX vs. O-MTX, P<0.001; SC-MTX vs. SWITCH, P=0.0094), and at 12 and 24 weeks later, the SC-MTX group exhibited significantly lower values than the O-MTX group (12 weeks, P<0.001; 24 weeks, P=0.0092). The P-values for the comparison between the other groups comparison at 24 weeks exhibited no significant differences (0.4155 for O-MTX vs. SWITCH and 0.7221 for SC-MTX vs. SWITCH). Although the disease activity trends using DAS28-CRP for each group are illustrated in Fig. 1, the remission rates based on the DAS28-CRP are illustrated in Figs. 2 and 3, and Tables SIII and SIV. As illustrated in Figs. 2 and 3, the initial remission rates were 20.30, 0, and 12.00% for the O-MTX, SC-MTX and SWITCH groups, respectively, indicating the lack of remission cases in the SC-MTX group at the start. Conversely, among cases with a high disease activity at introduction, 30 out of the 59 cases (50.848%) were noted in the SC-MTX group (data not shown). As demonstrated in Fig. 3, a significant difference in the initial remission rates was observed among the three groups using Fisher's exact test (P<0.001), and significant difference was shown between O-MTX and SC-MTX by pair-wise comparison (P<0.001) (Fig. 3). However, remission rates were noted at 4 weeks after starting treatment among the three groups (P=0.214). After 12 weeks, significant differences in remission rates were found among the three groups (P=0.0102) and a significant difference was found between O-MTX and SC-MTX by pair-wise comparisons (P=0.0054) (Fig. 3), suggesting higher remission rates in the SC-MTX group than in the other groups. Based on the aforementioned findings, the HAQ scores suggested that the SC-MTX group, including the SWITCH group, may achieve remission earlier than the O-MTX group. By contrast, despite the higher DAS28-CRP activity at introduction in the SC-MTX group than in the O-MTX group, early remission could still be achieved in many cases.

### Stratification based on anti-CCP antibody positivity may reveal differences in responsiveness according to the MTX administration route

**Figure 4:**
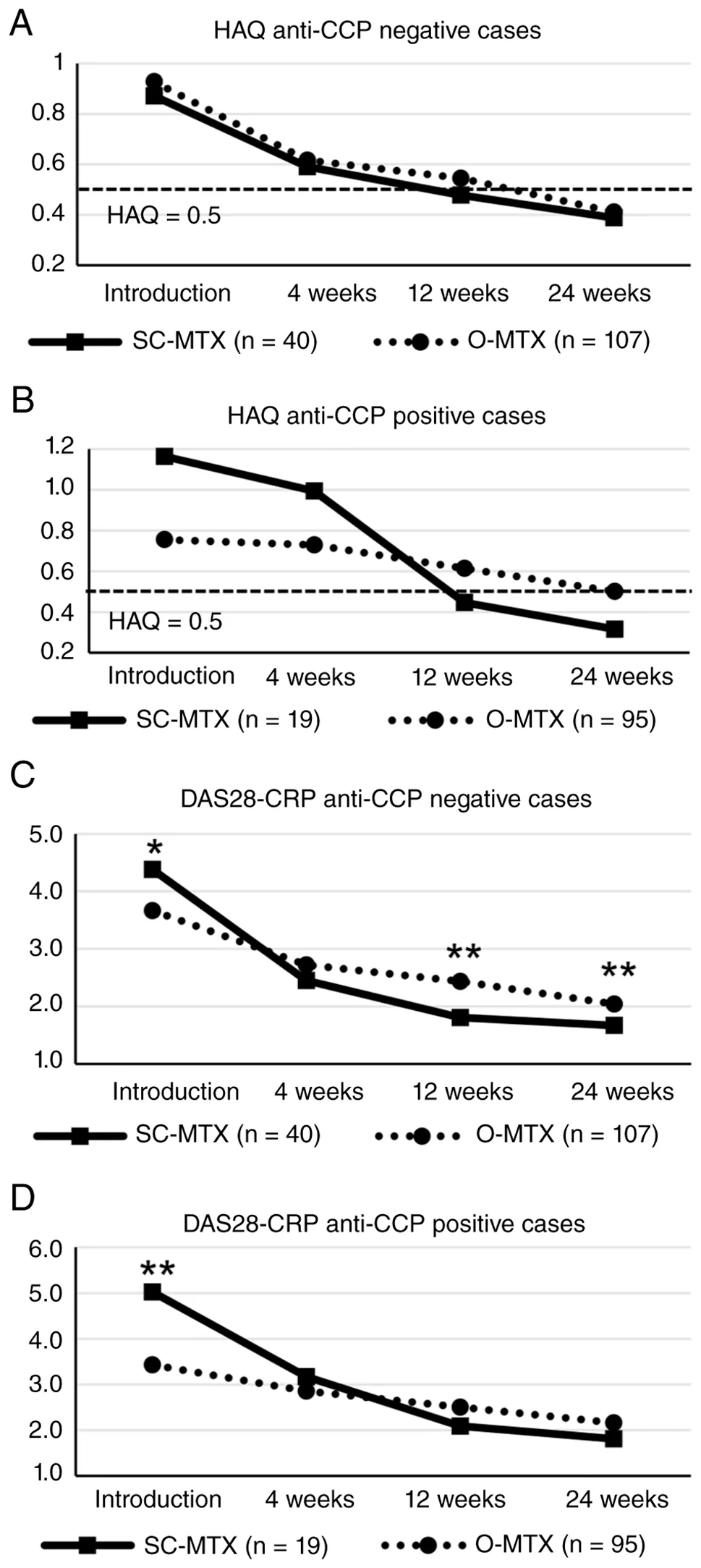
Comparison of changes in HAQ and DAS28-CRP scores based on anti-CCP positivity. (A and B) Transition of HAQ. The remission threshold for HAQ (0.5) is presented as a thin horizontal dotted line at the 0.5 mark. (C and D) Transition of DAS28-CRP. ^*^P<0.05 and ^**^P<0.0001, significant differences between two groups. HAQ, health assessment questionnaire; DAS28-CRP, disease activity score in 28 joints with C-reactive protein; CCP, cyclic citrullinated peptide; MTX, methotrexate; O-MTX, oral MTX administration; SC-MTX, MTX subcutaneous injection from the initial induction; W, weeks.

Subsequently, the SC-MTX and O-MTX groups were divided into anti-CCP antibody-negative and anti-CCP antibody-positive subgroups, and the changes in HAQ and DAS28-CRP were examined between the SC-MTX and O-MTX groups. As illustrated in Fig. 4, no significant difference in HAQ was observed between the SC-MTX and O-MTX groups, irrespective of the anti-CCP antibody status (negative or positive). On the one hand, when stratified by anti-CCP antibody status, in the antibody-negative group, the DAS28-CRP value was significantly higher in the SC-MTX than in the O-MTX group at the time of MTX introduction. However, at 12 and 24 weeks, the DAS28-CRP values were significantly lower, exhibiting an opposite trend to that observed at the initial MTX introduction. On the other hand, similar to that in the antibody-negative group, the DAS28-CRP value in the antibody-positive group was significantly higher in the SC-MTX than in the O-MTX group at the time of MTX introduction. However, no significant difference was observed in the DAS28-CRP values between the groups after 4 weeks. These results suggest that treatment responsiveness may vary depending on both the MTX administration route and anti-CCP antibody status.

### The initial introduction of SC-MTX results in a low concomitant use rate of biological agents and JAK inhibitors after 24 weeks, a high remission rate with monotherapy, and a high glucocorticoid DR

The data for the concomitant use rate of biological agents and JAK inhibitors, the remission rate with monotherapy, and glucocorticoid DR 24 weeks after SC-MTX or O-MTX introduction are presented in Table II. The O-MTX group had a higher concomitant use rate of biological agents and JAK inhibitors than the other groups, and the remission rate with MTX monotherapy was higher in the SC-MTX group than in the other groups. The SC-MTX group also exhibited a higher glucocorticoid DR than the other groups. These results suggest that the early SC-MTX introduction is a simple and effective treatment without the need for multiple drugs.

### In the O-MTX group, early SC-MTX introduction and SWITCH groups, no significant differences are observed in the trends of liver function, lipids, kidney function and blood cell counts

**Figure 5:**
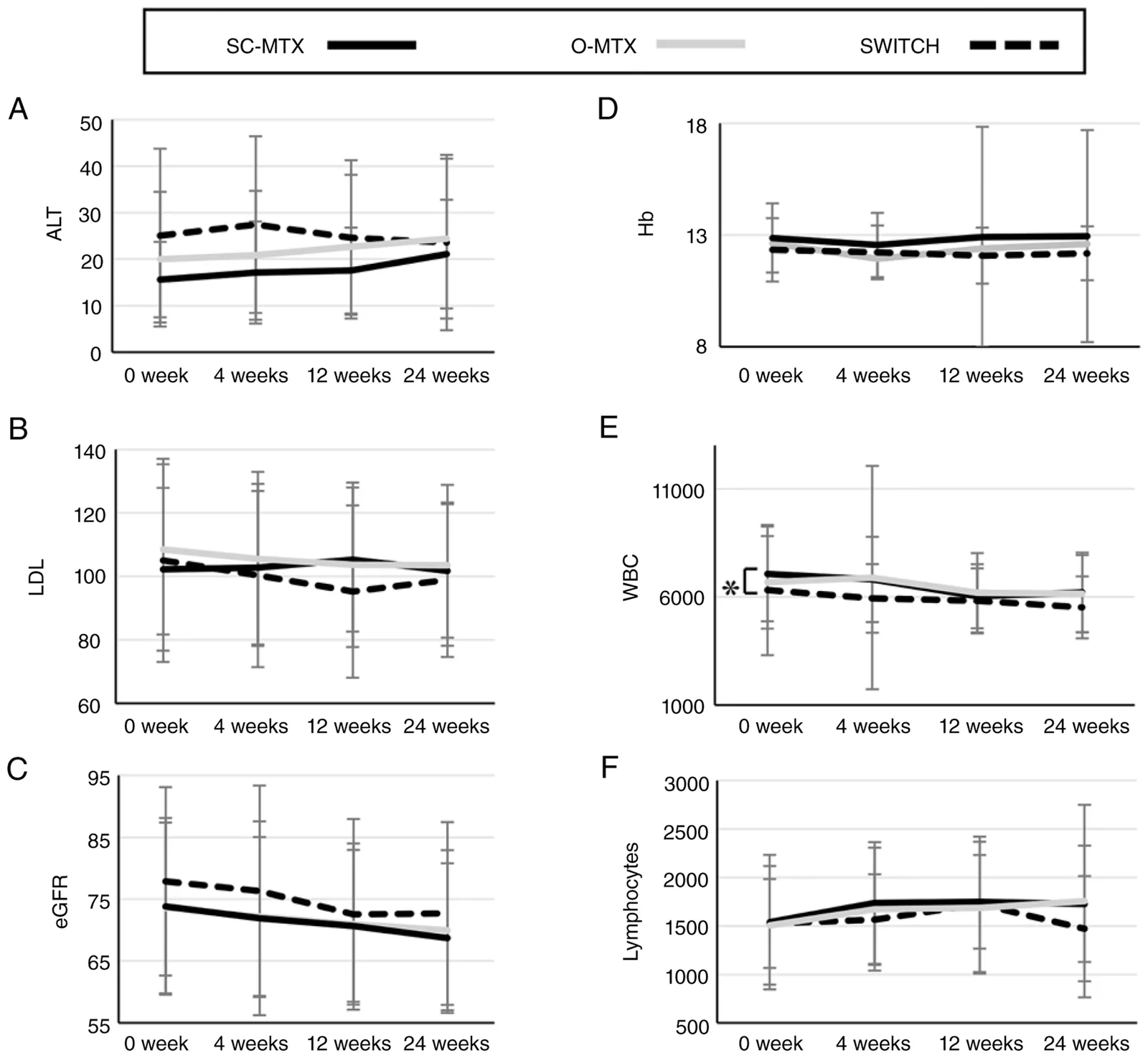
Changes in liver function, lipids, and blood cells over a 24-week period in the 3 groups. (A) ALT in U/l, (B) LDL in mg/dl, (C) eGFR in ml/min/1.73 m^2^, (D) Hb in g/dl, (E) white blood cell count in cells/µl, and (F) lymphocyte count in cells/µl. ^*^P<0.05, significant differences between the groups. ALT, alanine aminotransferase; Hb, hemoglobin; LDL, low-density lipoprotein; eGFR, estimated glomerular filtration rate; MTX, methotrexate; O-MTX, oral MTX administration; SC-MTX, MTX subcutaneous injection from the initial induction; SWITCH, switched group from O-MTX to MTX subcutaneous injection; W, weeks. ALT, alanine aminotransferase; LDL, low-density lipoprotein; eGFR, estimated glomerular filtration rate; Hb, hemoglobin; WBC, white blood cell count.

The lack of data on the changes in liver function, lipids, or blood cells during SC-MTX administration in Japan also required investigation. The data for the progression of ALT, LDL, eGFR and Hb levels and white blood cell and lymphocyte counts in the peripheral blood over 24 weeks from treatment initiation are presented in Fig. 5 and Table SII. Apart from a significant difference in the white blood cell count at baseline between the SC-MTX and SWITCH groups (P=0.046), no other significant differences were observed between the groups for any parameters at any time point. Conversely, when comparing eGFRs before the initiation and 24 weeks later, the eGFRs were 73.40±13.91 before and 69.74±13.67 after in the O- MTX group, 73.61±14.37 before and 68.34±12.34 after in the SC-MTX group, and 77.87±15.23 before and 72.66±14.77 after in the SWITCH group. In the O-MTX and SC-MTX groups, the eGFR was significantly lower at 24 weeks than that before the initiation (O-MTX: Introduction vs. 24 weeks; P=0.0081, SC-MTX: Introduction vs. 24 weeks; P=0.0473, SWITCH: Introduction vs. 24 weeks; P=0.2307). These results suggest that whether using O-MTX or SC-MTX, liver function, lipids, kidney function and blood cell systems exhibited similar trends in all three groups. However, caution should be exercised with respect to the decline in kidney function in both O-MTX and SC-MTX groups.

### No significant association exists between the cumulative dose of MTX and the change in eGFR, regardless of the administration route (oral or subcutaneous)

**Figure 6:**
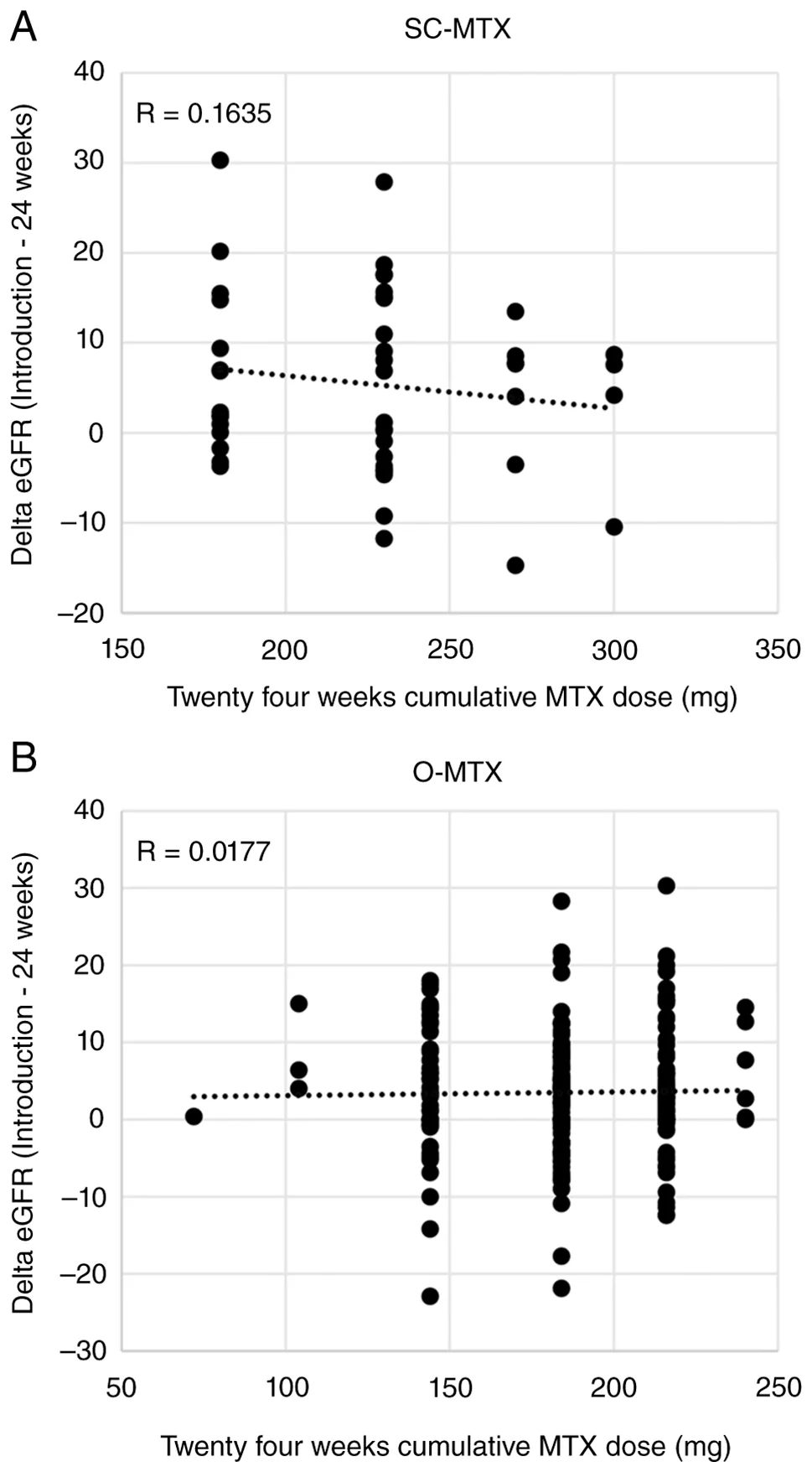
Association between cumulative MTX dose and change in eGFR. (A and B) The change in eGFR was calculated by subtracting the eGFR value at 24 weeks from the eGFR value at the start of MTX administration. (A) SC-MTX case, (B) O-MTX case. The straight line in the figure represents the regression line derived from the multiple regression analysis, and the R value indicates the correlation coefficient from that analysis. eGFR, estimated glomerular filtration rate; MTX, methotrexate; O-MTX, oral MTX administration; SC-MTX, MTX subcutaneous injection from the initial induction.

The present study then determined whether an association exists between the cumulative dose of MTX administered orally and subcutaneously and the change in eGFR 24 weeks after MTX administration compared with the time of MTX introduction. As demonstrated in Fig. 6, no significant association was observed between the cumulative dose of MTX and changes in eGFR in either the SC-MTX or O-MTX group. These results suggest that, over a 24-week period, variations in cumulative MTX dose are not associated with clinically significant changes in renal function.

### Despite some burden of drug costs, satisfaction with the SC-MTX pen is high

**Figure 7:**
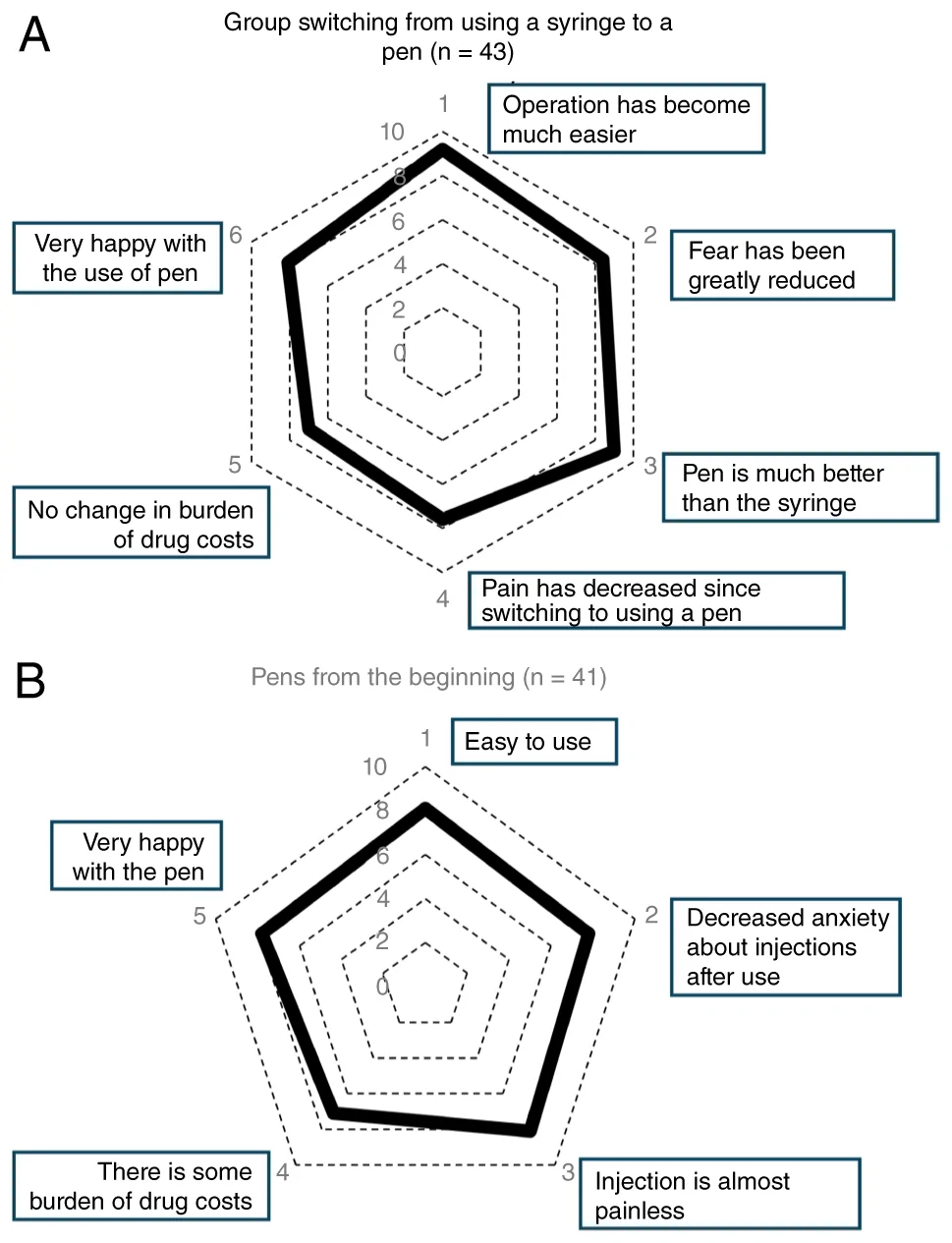
Results of patient satisfaction survey with the pen-type SC-MTX. (A) Survey results for cases where self-injection was changed from a syringe to a pen-type autoinjector. (B) Survey results for cases where a pen-type autoinjector was used from the beginning. In both cases, the highest satisfaction level was scored 10 points, and the average score for each case is shown with a bold line.

A patient questionnaire survey regarding the SC-MTX pen was conducted, which focused on changes in operation, pain and anxiety about injections, burden of drug costs, and overall satisfaction with the use of a pen (Fig. 7). The participants were divided into a group of 43 patients who switched from using a syringe to the SC-MTX pen and a group of 41 patients who had used the SC-MTX pen from the beginning. When rated on a scale of 10 points, with the higher score being better, regardless of age or sex, the burden of drug costs was rated an average of 7 points. However, all other participants rated an average of ≥8 points, indicating high satisfaction with the SC-MTX pen. Thus, satisfaction with the operation and other aspects of use would be high due to the effectiveness of the device, which is the reason for the high continuation rate.

## Discussion

In the present study, the SC-MTX group had a higher mean age than the other groups. However, Braun *et al* (8) reported that SC-MTX is more effective than O-MTX. Furthermore, elderly-onset RA is typically more active at disease onset than young-onset RA (YORA) (9). Therefore, in the present study, patients receiving SC-MTX were likely older than those administered O-MTX in their daily clinical practice. This represents a selection bias in clinical research; however, it is a limitation of the present study, as the present study was a retrospective analysis based on real-world clinical practice. The group that initiated treatment with SC-MTX had a higher remission rate when using a single agent than O-MTX or switching from O-MTX, a higher DR of glucocorticoids, and an earlier reduction in disease activity based on DAS28-CRP and HAQ scores, which had a significant effect on the improvement of the condition after 24 weeks. The usefulness of using SC-MTX early rather than the oral drug has been widely reported overseas. A 2019 meta-analysis from Boston University also reported that despite the lack of difference in the risk of adverse events, parenteral MTX therapy was significantly more likely to achieve a reduction in disease activity than O-MTX therapy (6). In addition, a 2015 report from China compared the two, demonstrated that subcutaneous MTX therapy may have an earlier therapeutic effect (5). Both reports utilized higher MTX doses than those used in Japan, which somewhat diverges from the actual clinical situation in Japan, and the present study is notable, as it presents a comparative analysis within actual clinical practice. There are reports that switching from O-MTX to SC-MTX improves disease activity and may reduce or delay the need for biologic therapy (10). However, to the best of my knowledge, no reports have directly compared group that initially received SC-MTX with the SWITCH group. Furthermore, no studies have examined the DR of glucocorticoids with the use of O-MTX and injectable formulations, and the present study also demonstrated that the DR of glucocorticoids was higher in the SC-MTX group. As regards the association between MTX administration and renal function decline, Hayashi *et al* (11) reported that in long-term administration (1 and 3 years), the greater the MTX dose, the greater the eGFR decline. However, that study did not examine the 6-month period or cumulative dose (11). Taken together with the findings of the present study, the results suggest that there is no clear association between cumulative MTX dose and the change in eGFR, at least in short-term observations.

The discontinuation rates due to stomatitis and anorexia were the same in the O-MTX and SC-MTX groups. In the present study, 31.7% of the patients who continued O-MTX therapy were prescribed vitamins and mouthwash to address stomatitis and antiemetics for anorexia. However, patients expressed reluctance to increase their dose due to fear of side-effects and preferred not to do so (unpublished observation). Previous studies have reported that the emergence of stomatitis and gastrointestinal symptoms during treatment is a major reason for discontinuing treatment (12,13) and this has been a major obstacle to using or increasing the dose of O-MTX until now. With SC-MTX, these symptoms are rarely observed, so the dose can be easily increased. In addition, even with increased doses, the frequency of injections does not increase, and the injection dose does not change significantly, which is thought to reduce the burden on patients (unpublished observation). In general, SC-MTX results in fewer symptoms, such as stomatitis and anorexia, and patients are willing to increase the SC-MTX dose if the disease activity improves (unpublished observation). Although the burden of drug costs may increase slightly, the liquid volume does not change significantly, and the frequency of injections once a week remains constant, so the SC-MTX dose can be increased more smoothly than with O-MTX (unpublished observation). Therefore, the ability to administer sufficient amounts of SC-MTX as a phase 1 MTX treatment for RA was suggested to influence the improvement in disease activity.

Early treatment with SC-MTX is already in circulation overseas, with studies reporting that it is more effective and has a higher continuation rate than O-MTX (14,15). This may be attributed to the high long-chain MTX-PG concentration, which is associated with disease activity and an increased intracellular MTX retention, resulting in accumulation, which increases drug availability and effectiveness. SC-MTX has a higher bioavailability rate (the rate at which the drug is utilized in the body) (16-20). In the present study, the use of SC-MTX was significantly higher than that of O-MTX. Given the high bioavailability of SC-MTX, administering sufficient amounts of SC-MTX may lead to reductions in the dose of glucocorticoids, achieve a high remission rate with a single drug, and further reduce disease activity at an earlier stage. Taken together with the findings of previous reports (16-20), the SWITCH group in the present study likely included patients whose disease activity was not adequately controlled with O-MTX, as well as those unable to receive an adequate dose due to adverse effects. The additional reduction in disease activity observed after switching to SC-MTX may be explained by its higher bioavailability and lower incidence of side-effects compared with O-MTX, which may allow patients to tolerate and maintain a more effective dose. Overseas reports have demonstrated that the safety of injectable formulations is equivalent to that of oral formulations, even though they can be administered in larger amounts (15). Furthermore, another study reported a high continuation rate (5). The dose of injectable formulations could be increased more easily than that of oral formulations, yet the safety and effectiveness can be expected to be the same.

In the present study, a questionnaire survey was conducted on patients regarding the SC-MTX pen, which revealed that the group that switched from using a syringe to a pen was highly satisfied with the ease of use. Even in the group that used the pen from the beginning, despite the initial anxiety about injections, the ease of use and minimal pain caused by the pen greatly reduced the sense of burden after the injection. Overseas, the results of a survey of 189 patients and medical professionals regarding the SC-MTX pen revealed the following points: Simple injection, small injection volume and short injection time (<5 s) (21-25). Notably, the questionnaire survey used herein did not compare satisfaction levels between O-MTX and SC-MTX. In other words, the results obtained from the questionnaire survey do not indicate the superiority of SC-MTX over oral MTX, but instead suggest that it is a more convenient administration method.

The present study has certain limitations that need to be acknowledged. First, based on the guidelines of the Japan College of Rheumatology, the initial dose of MTX is 6-8 mg (7). However, in Japan, the available dose for oral MTX is 2 mg per tablet, whereas the minimum dose for subcutaneous MTX is 7.5 mg, increasing in increments of 2.5 mg. Therefore, comparing data with exactly the same dosage in the present study was not feasible. In clinical studies, such as phase 3 trials, intention-to-treat (ITT) analysis is generally employed, where the study proceeds according to the treatment plan. When per-protocol (PP) analysis is used to verify the pure effect of ‘the treatment itself’, it is ideal to include only subjects who completed the treatment as planned. In fact, the PLANETRA trial incorporated ITT and PP analyses (26). However, the present study had a retrospective nature, and the analysis was limited to analyzable cases, rendering it infeasible to conduct the analysis using an ideal clinical research model. This should be considered a study limitation. The present study, in which the O-MTX and SC-MTX groups were divided into the anti-CCP antibody-positive and anti-CCP antibody-negative subgroups and time-series analysis of HAQ and DAS28CRP was conducted, demonstrated that the clinical response to SC-MTX may differ depending on the anti-CCP antibody status. However, given the limited sample size and the retrospective nature of the present study, these observations should be considered exploratory.

In conclusion, the drug cost for SC-MTX may be more burdensome than that for O-MTX; however, early improvement in disease activity and a high remission rate with a single drug may lead to a reduction in long-term drug costs. The present study suggests that the early introduction of SC-MTX is beneficial to all patients with RA. However, the early introduction of SC-MTX is particularly significant for patients with a high disease activity and should be considered.

## Supplementary Material

Changes in DAS28-CRP and HAQ among the three groups.

Summary of statistical analysis of clinical data among three groups based on the P-values.

Changes in disease activity in each group.

Changes in remission rate for each group.

## Figures and Tables

**Table I:** Discontinuation rate of MTX and treatment rate of stomatitis among the three groups.

	Group		
Items/parameters	O-MTX	SC-MTX	SWITCH
Initial no. of patients	237	69	35
No. of patients with discontinuation	35	10	10
No. of patients continuing MTX	202	59	25
Age, mean ± SD	62.0±13.2	69.1±10.5	57.4±14.4
Female, %	71.8	67.8	67.8
P-values (for comparisons of age)	O-MTX vs. SC-MTX: P<0.001		
	O-MTX vs. SWITCH: P=0.2722		
	SC-MTX vs. SWITCH: P<0.001		
Discontinuation rate, %	14.77	14.49	28.57
No. of patients following discontinuation	202	59	25
MTX dose at 24 weeks, mg/week, mean ± SD	8.32±1.74	10.00±2.23	10.40±2.67
P-values (for comparisons of MTX dose at 24 weeks)	O-MTX vs. SC-MTX: P<0.001		
	O-MTX vs. SWITCH: P<0.001		
	SC-MTX vs. SWITCH: NS		
Anti-CCP antibody-positive rate at the introduction of MTX, %	46.53	32.20	64.00
P-value (for comparisons of Anti-CCP antibody-positive rate)	P=0.0209		
Reason to switch to SC-MTX from O-MTX (n=25)			
Strengthening treatment (difficult to switch to bio/JAKi)			16 (64.00%)
Mouth ulcers and nausea			6 (24.00%)
Liver dysfunction			3 (12.00%)
Reasons for discontinuing MTX within 24 weeks (% per initial no. of patients)	n=35 (14.77%)	n=10 (14.49%)	n=10 (28.57%)
Ineffective	10 (4.22%)	1 (1.45%)	4 (11.43%)
stomatitis	3 (1.27%)	1 (1.45%)	2 (5.71%)
Anorexia	7 (2.95%)	1 (1.45%)	1 (2.86%)
Diarrhea		1 (1.45%)	
Renal dysfunction	8 (3.38%)		1 (2.86%)
Liver dysfunction	3 (1.27%)		1 (2.86%)
Pneumonia		1 (1.45%)	
Decreased blood count and lymph node swelling	1 (0.42%)	1 (1.45%)	1 (2.86%)
Discomfort with injections		2 (2.90%)	1 (2.86%)
Skin rash		1 (1.45%)	1 (2.86%)
Transfer to another hospital	3 (1.27%)	1 (1.45%)	1 (2.86%)
No. of patients who received medication for stomatitis and gastrointestinal symptoms	n=202	n=59	n=25
Medication for stomatitis and gastrointestinal symptoms, With/without symptoms (% with symptoms)	64/138 (31.68%)	6/53 (10.17%)	5/20 (20.00%)
P-value	O-MTX vs. SC-MTX: P=0.001		

MTX, methotrexate; O-MTX, oral MTX administration; SC-MTX, MTX subcutaneous injection from the initial introduction; SWITCH, switched group from O-MTX to SC-MTX; CCP, cyclic citrullinated peptide.

**Table II:** Rates of combined use of MTX with biological DMARDS and JAK inhibitors, remission with MTX alone, and glucocorticoid discontinuation after 24 weeks among the three groups.

	Group		
Items/parameter	O-MTX (n=202)	SC-MTX (n=59)	SWITCH (n=25)
Bio/JAK adoption rate within 24 weeks (with/without adoption)	32.18% (65/137)	20.34% (12/47)	24.00% (6/19)
P-value	P=0.1788		
Items/parameter	n=131	n=47	n=18
MTX alone case rate among remission cases at 24 weeks (MTX alone/not MTX alone in remission case)	31.30% (41/90)	65.96% (31/16)	33.33% (6/12)
P-value	P<0.001		
Items/parameter	n=131	n=47	n=18
Glucocorticoid discontinuation rate	59.3%	85.7%	50%

MTX, methotrexate; O-MTX, oral MTX administration; SC-MTX, MTX subcutaneous injection from the initial induction; SWITCH, switched group from O-MTX to SC-MTX.

## Data Availability

The data generated in the present study may be requested from the corresponding author.
