# Supplementary material for: Methotrexate subcutaneous injection for the initial treatment of rheumatoid arthritis can achieve earlier remission compared with oral administration
Source: Med Int (Lond). 2026 Aug 11;6(5):52. doi: 10.3892/mi.2026.336 (PMC13531110; doi:10.3892/mi.2026.336)
Supplement: Supplementary file 1 [file Supplementary_Data.pdf]

Table SI. Changes in DAS28-CRP and HAQ among the three groups.

	Group		
Items/parameter	O-MTX (n=202)	SC-MTX (n=59)	SWITCH (n=25)
DAS28-CRP			
Introduction	3.56±1.56	4.54±1.45	3.69±1.66
4 weeks	2.78±1.26	2.70±1.29	2.47±1.25
12 weeks	2.46±1.10	1.87±0.71	2.28±0.95
24 weeks	2.10±0.91	1.69±0.60	1.87±0.77
HAQ			
Introduction	0.84±1.84	0.98±0.87	0.75±0.83
4 weeks	0.670±0.67	0.72±0.71	0.56±0.71
12 weeks	0.57±0.58	0.46±0.49	0.40±0.59
24 weeks	0.45±0.54	0.35±0.51	0.43±0.63

DAS28-CRP, disease activity score in 28 joints with C-reactive protein; HAQ, health assessment questionnaire; MTX, methotrexate; O-MTX, oral MTX administration; SC-MTX, MTX subcutaneous injection from the initial induction; SWITCH, switched group from O-MTX to MTX subcutaneous injection.

Table SII. Summary of statistical analysis of clinical data among three groups based on the P-values.

	Compared group	Introduction	4 weeks	12 weeks	24 weeks
eGFR	SC-MTX vs. SWITCH<br>SC-MTX vs. O-MTX<br>SWITCH vs. O-MTX	0.4848	03878	0.8580	0.4656
AST	SC-MTX vs. SWITCH	0.0768	0.1369	0.1504	0.2123
	SC-MTX vs. O-MTX	0.0517	0.0589		
	SWITCH vs. O-MTX	0.5811	0.5642		
LDL	SC-MTX vs. SWITCH<br>SC-MTX vs. O-MTX<br>SWITCH vs. O-MTX	0.4527	0.4116	0.2133	0.8275
Hb	SC-MTX vs. SWITCH	0.1723	0.9908	0.0537	0.0969
	SC-MTX vs. O-MTX		0.0832		
	SWITCH vs. O-MTX		0.2256		
WBC	SC-MTX vs. SWITCH	0.0458	0.0559	0.8046	0.3362
	SC-MTX vs. O-MTX	0.1380			
	SWITCH vs. O-MTX	0.1521			
Lymph	SC-MTX vs. SWITCH<br>SC-MTX vs. O-MTX<br>SWITCH vs. O-MTX	0.9588	0.3763	0.3981	0.3857

The Kruskal-Wallis test was performed for 3 group comparisons. If the test revealed significance, the Steel-Dwass test was also used. AST, aspartate aminotransferase; eGFR, estimated glomerular filtration rate; Hb, hemoglobin; LDL, low-density lipoprotein; Lymph, lymphocyte count; MTX, methotrexate; O-MTX, oral MTX administration; SC-MTX, MTX subcutaneous injection from the initial induction; SWITCH, switched group from O-MTX to MTX subcutaneous injection; WBC, white blood cell count.

Table SIII. Changes in disease activity in each group.

A, O-MTX

Disease activity	Introduction	4 weeks	12 weeks	24 weeks
Remission	41 (20.3%)	80 (39.6%)	103 (51.0%)	132 (65.3%)
Low activity	22 (10.9%)	30 (14.9%)	23 (11.4%)	18 (8.9%)
Moderate activity	80 (39.6%)	69 (34.2%)	59 (29.2%)	46 (22.8%)
High activity	59 (29.2%)	23 (11.4%)	17 (8.4%)	6 (3.0%)
Total	202 (100%)	202 (100%)	202 (100%)	202 (100%)

B, SC-MTX

Disease activity	Introduction	4 weeks	12 weeks	24 weeks
Remission	0 (0%)	28 (47.5%)	43 (72.9%)	47 (81.0%)
Low activity	4 (6.8%)	7 (11.9%)	8 (13.6%)	6 (10.3%)
Moderate activity	25 (42.4%)	17 (28.8%)	7 (11.9%)	5 (8.6%)
High activity	30 (50.8%)	7 (11.9%)	1 (1.7%)	0 (0%)
Total	59 (100%)	59 (100%)	59 (100%)	59 (100%)

C, SWITCH

Disease activity	Introduction	4 weeks	12 weeks	24 weeks
Remission	3 (12.0%)	14 (56.0%)	13 (52.0%)	18 (72.0%)
Low activity	4 (16.0%)	1 (4.0%)	4 (16.0%)	0 (0%)
Moderate activity	12 (48.0%)	8 (32.0%)	7 (28.0%)	7 (28.0%)
High activity	6 (24.0%)	2 (8.0%)	1 (4.0%)	0 (0%)
Total	25 (100%)	25 (100%)	25 (100%)	25 (100%)

Data are presented as number and percentage. MTX, methotrexate; O-MTX, oral MTX administration; SC-MTX, MTX subcutaneous injection from the initial induction; SWITCH, switched group from O-MTX to MTX subcutaneous injection.

Table SIV. Changes in remission rate for each group.

	Remission rate (%)			
Group/period	Introduction	4 weeks	12 weeks	24 weeks
O-MTX remission/total (remission, %)	41/202 (20.3%)	80/202 (39.6%)	103/202 (51.0%)	132/202 (65.3%)
SC-MTX remission/total (remission, %)	5/59 (0%)	28/59 (47.5%)	43/59 (72.9%)	47/59 (81.0%)
SWITCH remission/total (remission, %)	3/25 (12%)	14/25 (56.0%)	13/25 (52.0%)	18/25 (72.0%)
Group comparison (Fisher's exact test) P-value	<0.001	0.214	0.0102	0.047
Multiple comparison (Marascullo's method)				
Compared group	P-value	P-value	P-value	P-value
O-MTX vs. SC-MTX	<0.001	N.A.	0.0054	0.9199
O-MTX vs. SWITCH	0.993	N.A.	0.9955	0.7963
SC-MTX vs. SWITCH	0.1819	N.A.	0.1949	0.7493

Multiple comparisons (Marascullo's method) were performed if the group comparison exhibited a significant difference. N.A., not analyzed. MTX, methotrexate; O-MTX, oral MTX administration; SC-MTX, MTX subcutaneous injection from the initial induction; SWITCH, switched group from O-MTX to MTX subcutaneous injection.
